# Extraction optimization, antioxidant activity, and tyrosinase inhibitory capacity of polyphenols from *Lonicera japonica*

**DOI:** 10.1002/fsn3.1021

**Published:** 2019-04-21

**Authors:** ZiLuan Fan, Lu Li, XiaoLin Bai, Hua Zhang, QiRui Liu, He Zhang, YuJie Fu, Rumbani Moyo

**Affiliations:** ^1^ School of Forestry Northeast Forestry University XiangFang, Harbin China; ^2^ Department of Food Science and Engineering, School of Chemistry and Chemical Engineering Harbin Institute of Technology Nangang, Harbin China; ^3^ Key Laboratory of Forest Plant Ecology, Ministry of Education Northeast Forestry University Harbin China

**Keywords:** antioxidants, *Lonicera japonica*, polyphenols, tyrosinase

## Abstract

The objective of this research was twofold: first, to optimize the extraction process of *Lonicera japonica* polyphenols using a response surface methodology, and second, to study the antioxidant activity and tyrosinase inhibitory capacity of the polyphenols of different purities. High‐speed shearing homogenization extraction was used to extract the polyphenols from *L. japonica*. The antioxidant activity and the effect of polyphenols on tyrosinase activity were studied using free radical scavenging assay and the tyrosinase method, respectively. The optimal extraction conditions with an extraction yield of 6.96% for polyphenols were determined as follows: ethanol volume fraction 57%, shearing time 3.30 min, and solid–liquid ratio 1:58. *Lonicera japonica* polyphenols exhibited potent scavenging activity on 1,1‐diphenyl‐2‐picrylhydrazyl (DPPH) and 2, 2'‐azino‐bis(3‐ethylbenzothiazoline‐6‐sulfonic acid) (ABTS), and inhibitory capacity on tyrosinase. The results suggested that *L. japonica* polyphenols could be explored as a natural antioxidant and tyrosinase inhibitor.

## 1 INTRODUCTION

The herb, *Lonicera japonica*, is a member of the Caprifoliaceae family and is regularly used in traditional Chinese medicine. Past research has isolated 140 organic compounds from *L. japonica*, including essential oils, flavonoids, organic acids, and triterpene glycosides (Kuroda, Shizume, & Mimaki, 2014; Shang, Pan, Li, Miao, & Ding, 2011). Recent studies have shown that extracts from *L. japonica* have a wide range of bioactive properties, such as anti‐inflammatory (Chen, Liou, Tzeng, Lee, & Liu, 2012), antibacterial (Xiong et al., 2013), and antioxidant use (Wang et al., 2014), and are used to treat nephrotic diabetes (Tzeng, Liou, Chang, & Liu, 2014) and metabolic diseases (Shin et al., 2017). Tyrosinases (EC1.14.18.1), one of the extracts from *L. japonica*, are metalloenzymes belonging to the type‐3 (binuclear) copper protein (Rolff, Schottenheim, Decker, & Tuczek, 2011) that exist widely in microorganisms (Faccio, Kruus, Saloheimo, & Thony‐Meyer, 2012), animals (Hu, Wang, Deng, & Jiang, 2016), and plants (Zekiri et al., 2014). Tyrosinases catalyze the ortho‐hydroxylation of tyrosine to dihydroxy‐phenyl‐alanine (DOPA) and the subsequent two‐electron oxidation to dopaquinone (Rolff et al., 2011). They are also related to enzymatic browning in fruit and vegetables, pigmentation, and insect physiological processes (Balabanidou, Grigoraki, & Vontas, 2018; Olmedo et al., 2018; Xue et al., 2018).

Common methods for polyphenols extraction include solvent extraction (Seo et al., 2012), enzymatic hydrolysis (Tang et al., 2018), microwave extraction (Nayak et al., 2015), and ultrasonic‐assisted extraction (Sousa et al., 2016). These methods often have an extensive process time, which results in partial destruction of the polyphenols structure and reduced extraction yield. High‐speed shearing homogenization extraction (high shear) is an emerging extraction technology, which has less process time, lower energy consumption, and lower extraction temperature with a highly efficient result. High shear is often used to extract polysaccharides, pectin, and other biologically active ingredients (Fan, Lin, Wang, & Wang, 2014; Guo et al., 2017). Some studies have demonstrated the role of polyphenols in inhibiting tyrosinase activity, but not the potential relationships between polyphenols purity and their inhibitory effect. In the present study, the extraction of polyphenols from the *L. japonica* flowers through high shear technology was optimized using response surface methodology together with a Box–Behnken design. The antioxidant activity and the effect of the extracted polyphenols on tyrosinase activity were studied.

## 2 MATERIALS AND METHODS

For this study, the *L. japonica* was harvested in Quzhou, Anhui province. They were then crushed through a 40‐mesh sieve. All chemicals, solvents, and analytical reagents such as deionized water, tyrosinase, l‐Dopa, and kojic acid were purchased from Baoman Biotechnology Co., Ltd. (Shanghai, China).

### 2.1 Total polyphenol yield

The total polyphenol yield was slightly modified based on Evstatieva, Todorova, Antonova, and Staneva (2010) method. A volume of 1.0 ml of Folin–Ciocalteu reagent was mixed with 1.0 ml of sample solution, 5.0 ml of distilled water, and 3 ml of sodium carbonate (15%). The mixture was then left to stand for 2 hr. Absorbance was then measured at a wavelength of 765 nm, and a standard curve linear regression equation was used to calculate the total polyphenol concentration and yield (Zheng et al., 2018). The extraction yield was calculated using the following formulation.

$$\%\text{Extraction}\;\text{yield}=\frac{C\times V\times \text{Dilution}\;\text{times}}{1.00\times 1,000}\times 100.$$

In the formula: C—Total polyphenol concentration in the extract sample, mg/ml; V—Fixed volume of extraction, ml.

$$\%\text{Purity}=\frac{\text{Total}\;\text{polyphenols}\;\text{mass}\;\text{in}\;\text{the}\;\text{sample}}{\text{Total}\;\text{sample}\;\text{mass}}\times 100.$$

### 2.2 Single factor experiment

To investigate the effect of solid–liquid ratio, 1.00‐g sample was added to a 60% ethanol solution using the ratios of 1:10, 1:20, 1:30, 1:40, 1:50, 1:60 (g/ml), and 3 min as shearing time. To investigate the effect of ethanol volume fraction, the following volume fractions: 20%, 30%, 40%, 50%, 60%, 70%, 80%, and 90% of ethanol were used. Solid–liquid ratio was 1:50 and the shearing time was 3 min. To investigate the effect of shearing time, 1, 2, 3, 4, and 5 min were used to extract polyphenols at a solid–liquid ratio of 1:50 and a 60% ethanol volume fraction.

### 2.3 Response surface assay

The extraction condition of the polyphenols was optimized by the response surface methodology, with the extraction yield of polyphenols as the response value. Ethanol volume fraction (A), shearing time (B), and solid–liquid ratio (C) were chosen as the independent variables.

### 2.4 DPPH free radical scavenging assay

The DPPH scavenging activity assay was conducted based on the Zheng, Lin, Su, Zhao, and Zhao (2015) method. A 2 ml sample solution with different contents was mixed with 2 ml of a DPPH ethanol solution (0.2 mM). The reaction mixture was then shaken and allowed to stand for 30 min in a dark environment. The absorbance was measured at a wavelength of 517 nm (*A*_i_). 70% ethanol solution was used as a reference and the absorbance was measured (*A*_j_), which followed the completed reaction of a 2 ml sample solution mixed with a 2 ml of 70% ethanol solution. The absorbance was measured as equal amount of 70% ethanol instead of sample as the blank (*A*_0_). With Trolox as a positive control, the DPPH radical scavenging rate was determined.

$$\%\text{Clearance}\;\text{rate}=\frac{A_{0}-A_{i}+A_{j}}{A_{0}}\times 100.$$

### 2.5 ABTS radical scavenging assay

The ABTS free radical scavenging activity was assayed using a method previously described by Marecek et al. (2017) with a slight modification. ABTS reaction solution was prepared by mixing 7 mM of ABTS aqueous solution with 2.45 mM of K_2_S_2_O_8_ solution in a dark room for 24 hr. Then, the ABTS stock solution was diluted with absolute ethanol to obtain an absorbance of 0.70 ± 0.02 at 734 nm. Then, 0.4 ml sample solution with different contents or Trolox standard was mixed with 3 ml of the ABTS solution. The mixture was then shaken and allowed to stand for 8 min in a dark environment. The absorbance, set at a 734 nm wavelength, was then measured (*A*_i_). For the control, absolute ethanol was used instead of ABTS. Then, the absorbance was measured as 0.4 ml distilled water instead of the sample as the blank (*A*_0_).

$$\%\text{Clearance}\;\text{rate}=\frac{A_{0}-A_{i}+A_{j}}{A_{0}}\times 100.$$

### 2.6 Determination of tyrosinase inhibitory capacity

Tyrosinase inhibition rate was determined using l‐Dopa as substrate (Paun, Neagu, Albu, & Radu, 2015). Briefly, 200 μl of sample solution and 200 μl of mushroom tyrosinase (0.5 mg/ml in 0.2 mM PBS buffer, pH 7.0) were mixed with 3,700 μl of PBS and pre‐incubated at 30°C for 15 min. Finally, the l‐Dopa (100 μl, 10 mM) was added to the mixture and the absorbance (*A*_i_) was measured at 475 nm after 3 min. The sample solution was replaced by an equal amount of PBS, and the absorbance (*A*_0_) was measured under the same conditions. Kojic acid was used as a positive control.

$$\%\text{Inhibition}\;\text{rate}=\frac{A_{i}-A_{0}}{A_{i}}\times 100.$$

### 2.7 Statistical analysis

All measurements were done in triplicate and the results were expressed as mean value ± standard deviation (*n* = 3). The data between the control and experimental groups were analyzed by *p*‐values. *p*‐values <0.05 were considered as significant. To obtain the best extraction process, ANOVA and 3D surface maps of the model (Table 1) were used to analyze the experimental and predictive data to determine the accuracy of the model.

**Table 1:** Box–Behnken design matrix for optimization of parameters and the response values for the extraction yield of polyphenols. Ethanol volume fraction (A), shearing time (B), and solid–liquid ratio (C)

No.	A/%	B/min	C/(g/ml)	Extraction yield/%
1	30	1	1:50	6.48142
2	70	1	1:50	6.48142
3	30	5	1:50	6.39549
4	70	5	1:50	6.55017
5	30	3	1:30	5.87986
6	70	3	1:30	5.84548
7	30	3	1:70	5.87985
8	70	3	1:70	6.75643
9	50	1	1:30	5.86267
10	50	5	1:30	6.18923
11	50	1	1:70	6.67049
12	50	5	1:70	6.65330
13	50	3	1:50	6.87673
14	50	3	1:50	6.83267
15	50	3	1:50	6.90830
16	50	3	1:50	6.79080
17	50	3	1:50	6.82518

## 3 RESULTS

### 3.1 Single factor assay: Effect of solid–liquid ratio on polyphenol extraction yield

**Figure 1:**
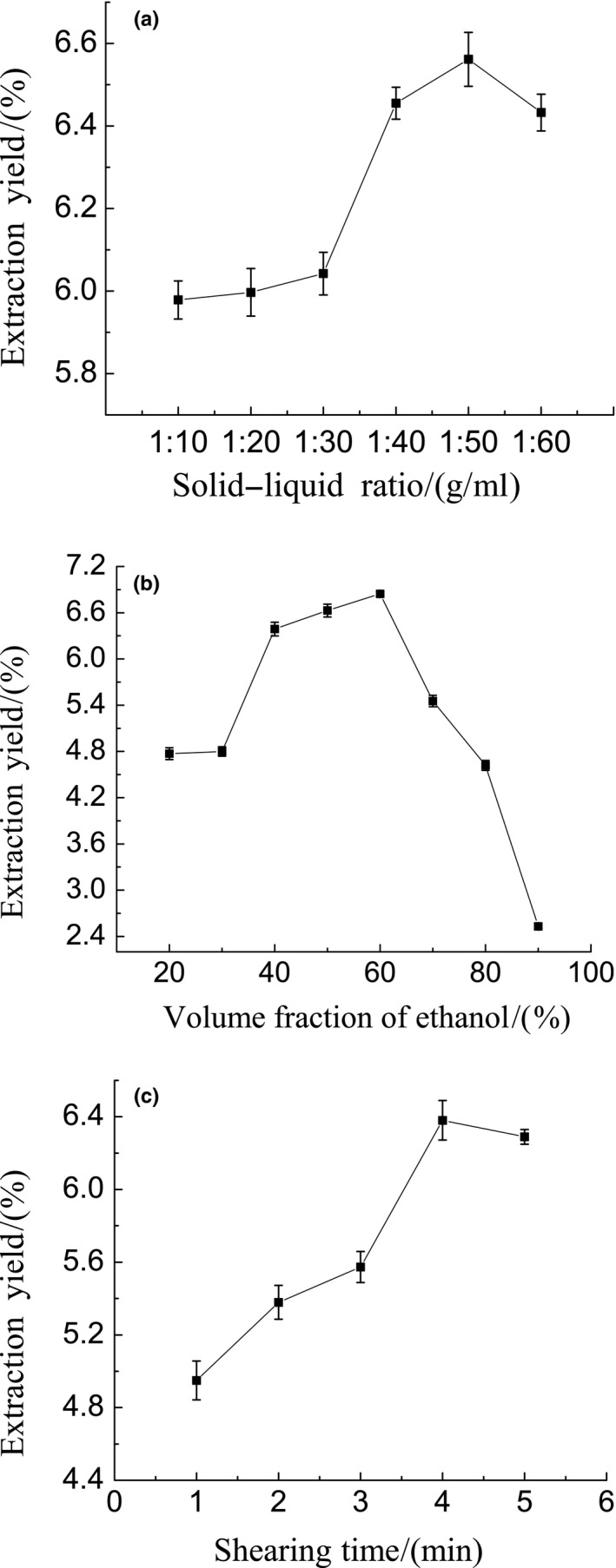
(a–c) Single factor assay: (a) Effect of solid–liquid ratio on the extraction yield of polyphenols. (b) Effect of ethanol volume fraction on the extraction yield of polyphenols. (c) Effect of shearing time on the extraction yield of polyphenols

Figure 1a shows the effect of solid–liquid ratio on the total polyphenol yield. The extraction yield increased gradually with increasing solid–liquid ratio with the maximum value being obtained at 1:50 (g/ml) ratio. The extraction yield remained low at ratios 1:10 to 1:30, but beyond 1:30 ratio, the extraction yield increased rapidly. The extraction yield decreased with the solid–liquid ratio decreasing from 1:50 to 1:60. 1:50 (g/ml) was chosen as the solid–liquid ratio.

### 3.2 Single factor assay: Effect of ethanol volume fraction on extraction

Figure 1b illustrates that an increase in ethanol volume fraction causes an increase in polyphenol extraction yield but the yield dwindles as the ethanol volume fraction increases further. The increase in extraction yield from 20% to 60% could be due to the high solubility of polyphenols in ethanol. When the ethanol volume fraction exceeds 60%, the yield of polyphenols decreases. For cost‐effective and easy ethanol recovery purposes, 50% ethanol volume fraction was selected.

### 3.3 Single factor assay: Effect of shearing time on total polyphenols yield

From Figure 1c, the extraction yield increased significantly with increasing shearing time and the maximum extraction yield was obtained at 4 min, but it declined after 4 min. Taking into account the establishment of the response surface model, 3 min was chosen as the inflection point.

### 3.4 Response surface assay

Design‐expert software (Design Expert 8.0.6; Stat‐Ease Inc.) was used for data statistical analysis. Regression analysis second‐order polynomial equation was developed to study the relationships between input process variables and respective responses：Extraction yield = 6.83 + 0.12A + 0.037B + 0.27C + 0.039AB + 0.23AC − 0.086BC − 0.30A_2_ − 0.051B_2_ − 0.44C_2_. The coefficient of determination is *R* ^2^ = 0.9542.

As illustrated in Table 2, the highly dominant factors that influenced the extraction of the total polyphenols were C, AC, A_2_, and C_2_, and the dominant factor was A. The coefficient of variation was 1.93, which indicated that the test was reliable. The signal‐to‐noise ratio (11.688, larger than 4) signified that the model had a strong response signal, and that the model explained the change to the 89.53% response value. This model fits well and is suitable for use to predict the extraction process of total polyphenols from *L. japonica*. By testing the regression coefficient of Y, the order of influencing factors on the extraction yield of polyphenols is C (solid–liquid ratio) > A (ethanol volume fraction) > B (shearing time).

**Table 2:** Analysis of variance (ANOVA) for extraction yield of polyphenols (Y) as a function of Ethanol volume fraction (A), shearing time (B), and solid–liquid ratio (C) used during extraction

Source	Sum of squares	Degree of freedom	Mean squares	*F*‐value	*p*‐value Prob > *F*
Model	2.27	9	0.25	16.20	0.0007^a^
A	0.12	1	0.12	7.98	0.0256
B	0.011	1	0.011	0.69	0.4349
C	0.60	1	0.60	38.27	0.0005
AB	5.981E‐003	1	5.981E‐003	0.38	0.5549
AC	0.21	1	0.21	13.33	0.0082
BC	0.030	1	0.030	1.90	0.2107
A_2_	0.39	1	0.39	25.10	0.015
B_2_	0.011	1	0.011	0.71	0.4288
C_2_	0.81	1	0.81	51.85	0.0002
Residual	0.11	7	0.016		
Lack of Fit	0.089	3	0.030	5.85	0.0605^b^
Pure Error	0.020	4	5.058E‐003		
Cor Total	2.38	16			

a Significant.

b Not significant.

**Figure 2:**
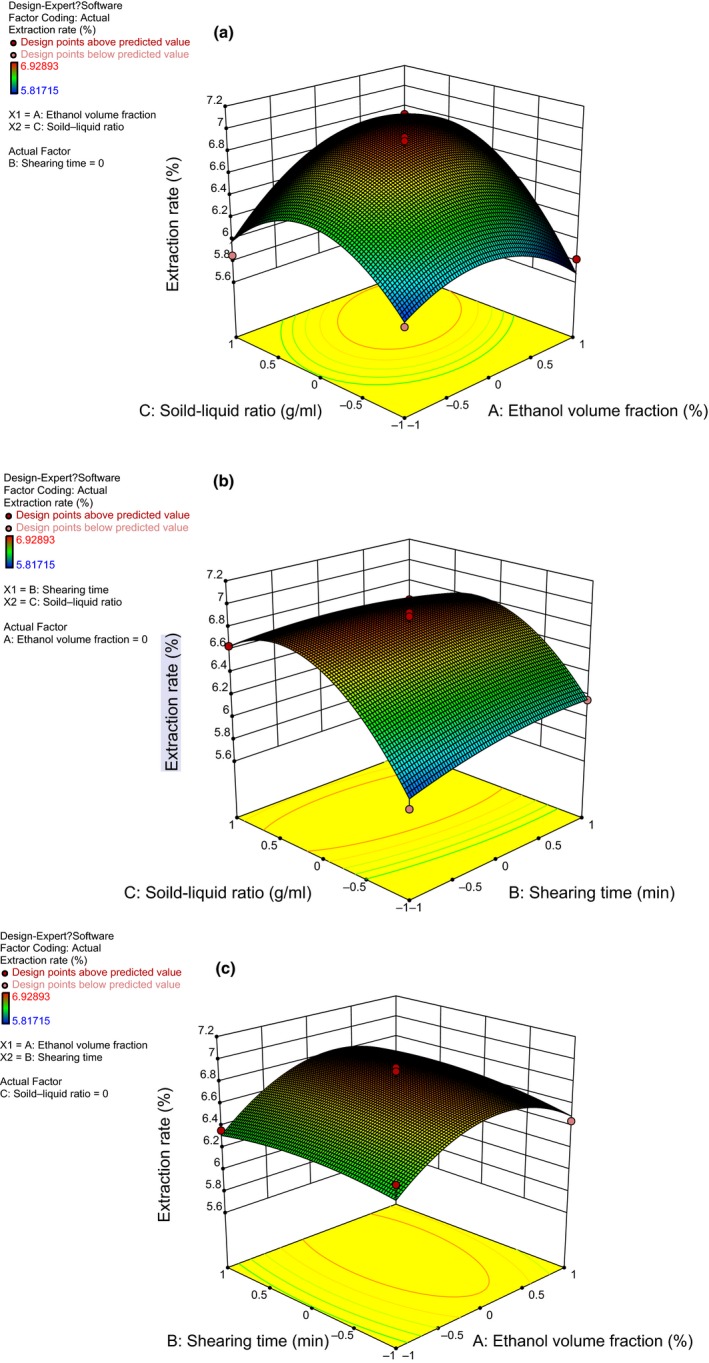
(a–c) Response 3D surface Logic: (a) Solid–liquid ratio, interaction of ethanol volume fraction and polyphenols extraction yield. (b) Shearing time, interaction of solid–liquid ratio on polyphenols extraction yield. (c) Volume fraction of ethanol, interaction of shearing time on polyphenols extraction yield

As illustrated in Figure 2a, when the shearing time settled, solid–liquid ratio and ethanol volume fraction increased the extraction yield at first but decreased later. Figure 2b shows that shearing time had a minor effect on the extraction yield, while solid–liquid ratio had an increasing effect at first which dwindled later. Figure 2c shows that extraction yield initially increased with the increase in ethanol volume fraction but decreased later with further increase in ethanol volume fraction, but shearing time had the same effect on the extraction yield. When taking into account the convenience of practical operation, and after further adjustment, the highest yield was achieved using 57% ethanol. Consequently, the extraction time became 3.30 min, with a 1:58 solid–liquid ratio.

### 3.5 DPPH free radical scavenging assay

**Figure 3:**
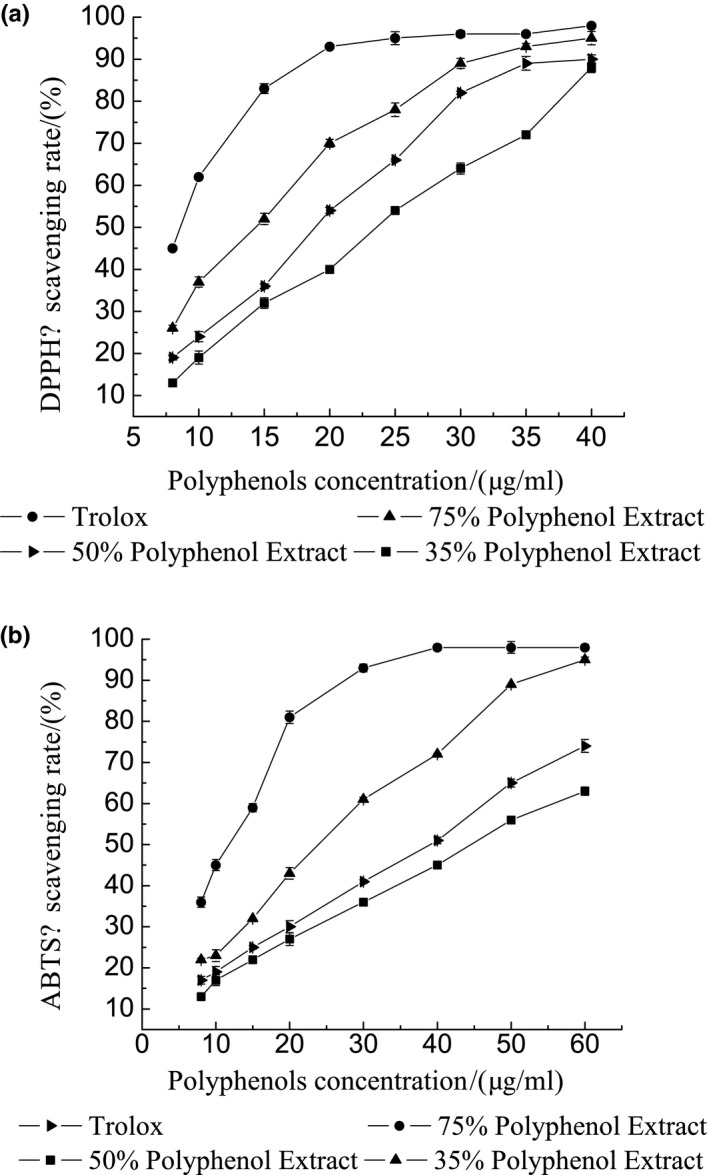
(a, b) Free radical scavenging assay: (a) The effect of the total polyphenols extraction on the scavenging of DPPH. (b) The effect of the total polyphenols extraction on the scavenging of ABTS

Historically, DPPH free radical scavenging assays are used to evaluate the activity of antioxidants, as it is a very stable free radical that can be stored for a long period. The DPPH free radical is an aromatic radical which has three aromatic ring structures (Guo et al., 2004). As shown in Figure 3a, the scavenging rate of DPPH increased as the polyphenols concentration increased from 15 to 40 μg/ml before it became stable. The IC_50_ of 75% (A), 50% (B), and 35% (C) of polyphenols purities was (13.643 ± 0.039), (19.179 ± 0.52), and (23.669 ± 0.55) μg/ml, respectively. The IC_50_ of Trolox in the control group was (8.136 ± 0.45) μg/ml. The order of scavenging power was Trolox > A > B > C.

### 3.6 ABTS radical scavenging assay

Figure 3b shows that increase in polyphenols concentration from 10 to 60 μg/ml increases ABTS radical scavenging effect in a dose‐dependent manner. The IC_50_ of 75% (A), 50% (B), and 35% (C) of the total polyphenols was (24.822 ± 0.161), (38.181 ± 0.059), and (45.238 ± 0.18) μg/ml, respectively. 75% (A) of the total polyphenols exhibited a significantly higher scavenging effect and at 60 μg/ml, A and Trolox had a similar scavenging capacity. The IC_50_ of Trolox in the control group was (11.690 ± 0.73) μg/ml. The order of scavenging power was Trolox > A > B > C.

### 3.7 Determination of tyrosinase inhibitory capacity

**Figure 4:**
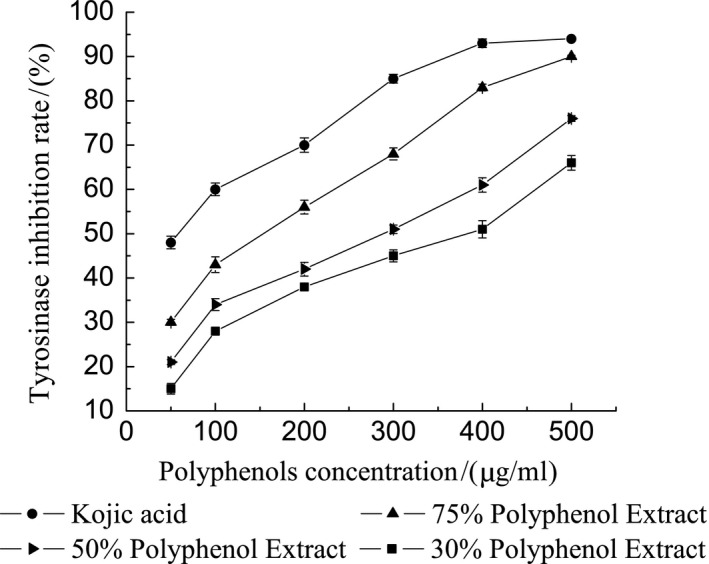
The effects of total polyphenols on the inhibitory ability of tyrosinase

Figure 4 shows that at the same concentration, kojic acid (IC_50_ = [54.920 ± 1.89] μg/ml) had the highest inhibition rate, followed by A (IC_50_ = [205.826 ± 0.23] μg/ml), B (IC_50_ = [282.229 ± 1.12] μg/ml), and C (IC_50_ = [355.270 ± 0.84] μg/ml). The order of inhibition ability was kojic acid > A > B > C.

## 4 DISCUSSION AND CONCLUSIONS

Our results showed that an increase in contact area of the solvent with *L. japonica* powder caused a decrease in material viscosity and an enhancement in the osmotic pressure and intermolecular driving force. As a result, polyphenols dissolved easily, which is consistent with the conclusions of Wong, Li, Li, Razmovski‐Naumovski, and Chan (2017) and Zhang Pechan and Chang (2018). The increase in ethanol content is beneficial to increase the solubility of phenolic compounds, increase the diffusion coefficient, lower the viscosity coefficient, and also reduce the interfacial tension in the pores. An increase in the proportion of ethanol reduces the dielectric constant of the solution, reducing the energy required to separate the solvent molecules, thereby allowing the solute molecules to enter the solid powder. However, when the solvent was increased further, the alcohol‐soluble components and impurities eluted together with the polyphenols and resulted in a decrease of the extraction yield. In addition, excessive solvent may cause insufficient force and a small number of cells fail to break the cell wall, resulting in a reduction in the dissolution of polyphenols.

In contrast, we also concluded that the extraction yield might be affected by the structure of polyphenols. Previous studies have classified polyphenols as flavonoids and nonflavonoid compounds; two main forms are as follows: glycosides and aglycone (Santhakumar, Battino, & Alvarez‐Suarez, 2018). In keeping with the principle of similar dissolution, overall polarity will enhance with the decrease of the ethanol mass fraction in a solution, which favors the solubility of polar substances. As the mass fraction of ethanol increases, the nonpolarity enhances and the amount of dissolved polar species decreases. Aglycone is a polar substance, so we speculate that *L. japonica* polyphenols might be polar isoflavones, which is similar to the result found by Wong et al. (2017). The yield of polyphenol compounds in less polar solvents is affected, thus reducing the solubility of polyphenolic compounds. *Lonicera japonica* polyphenols compounds mostly present in the form of glycosides. Generally, the glycoside‐containing polyphenols have good water solubility. When the ethanol concentration is low, the extraction rate will enhance with the increase in concentration. When the ethanol concentration continues to increase, the solubility of the glycoside gradually decreases, and the extraction rate gradually decreases. Glycosides are easily soluble in low‐concentration ethanol solutions, and aglycones are easily soluble in high‐concentration ethanol solutions. The results of this experiment indicate that the glycosides in the total polyphenols of *L. japonica* account for the majority. When the proportion of ethanol raises, increased interference factors fat‐soluble substances, reduced extraction rate. In addition, soluble polyphenols exist mainly in cells, whereas insoluble polyphenols are mostly deposited in cell walls to combine hydrogen bonds together with hydrophobic bonds with proteins and polysaccharides. A low proportion of ethanol in solvent is beneficial for polyphenol entrance into the cell. Furthermore, when the proportion of ethanol raises, the protein will denature on the cell wall, preventing the dissolution of polyphenols and reducing the extraction yield (d'Alessandro, Kriaa, Nikov, & Dimitrov, 2012).

The downward trend in extraction yield as shearing time increases can be explained by the following three reasons. Firstly, long‐term shearing time completely breaks the cell wall and other substances are released from the cytosol. This affects the osmotic pressure of the solvent and thus limits the solubility and permeability of the polyphenol, and interferes with the measurement result (Wong et al., 2017). Secondly, long‐term shear may cause damage to the cell wall and modify the polyphenols microstructure. Thirdly, excessively long shearing process generates heat, which increases oxidation.

There are many kinds of phenolic substances in *L. japonica* (Seo et al., 2012), including chlorogenic acid, caffeic acid, ferulic acid, coumaric acid, cinnamic acid, rutin, and luteolin. Its biological function is mainly attributed to the specific chemical structure of polyphenols. Due to the aromatic nature and highly conjugated systems with multiple hydroxyl groups, these compounds have beneficial electron or hydrogen atom donors, which neutralize free radicals and other reactive oxygen species. Each phenolic compound exhibits an antioxidant effect (Zhang et al., 2018), achieved by hydrogen supply and chelation of metal ions; an anticancer effect (Lee et al., 2016), primarily through antioxidant functions; the ability to prevent cardiovascular disease (Croft et al., 2018), which regulates blood lipid density in the blood, inhibits the oxidation of low‐density lipoprotein, and promotes vasodilation, thereby achieving cardiovascular protection.

A shearing time of 3.30 min, ethanol volume fraction of 57%, and a solid–liquid ratio of 1:58 were proven optimal in the case of the *L. japonica* polyphenols; the highest extraction yield was 6.96%. The results of DPPH, ABTS, and tyrosinase test were as follows: positive control (A) > 75% polyphenols extraction (B) > 50% polyphenols extraction (C) > 35% polyphenols extraction (D).

From the results, the purity of polyphenols increased both the antioxidant capacity and the ability of tyrosinases inhibition increased to varying degrees. However, the dose relationship of the three curves is not exactly the same. For example, when the concentration in Figure 3a was 35 μg/ml, the difference between the scavenging rates of B and C was significant. Moreover, when it was 40 μg/ml, the scavenging capacities of B and C were similar. However, the purity of B was 1.4 times that of C. This result signifies that during the purification process, different purity extraction may have different effective antioxidant components. The results of scavenging ABTS and inhibiting tyrosinases activity are similar to DPPH.

Benavente‐Garcia et al. demonstrated that the ability of polyphenolic compounds from olive leaves to scavenge ABTS radicals is affected by functional groups present in their structure, mainly B‐ring catechol, 3‐hydroxyl, and 3‐oxo function with 3 double‐bond conjugates (Hayes, Allen, Brunton, O'Grady, & Kerry, 2010). For other phenolic compounds, their ability to scavenge ABTS free radicals is primarily affected by the number and location of free hydroxyl groups in the structure. This indicates that the polyphenolic compound contained in A in Figure 3b has more phenolic hydroxyl groups than B and C.

As shown in the Figure 3a, *L. japonica* polyphenols have DPPH free radical scavenging activity in different purity. When the concentration was 10 μg/ml, the purity of the three different elution did not reach 50%, the clearance of 75% elution purity was 38.10%, and the purity of 35% elution only reached 13.90%. At this time, Trolox's clearance rate reached 44.65%. When the concentration reached 25 μg/ml, the clearance rate reached more than 50%, and the 75% elution purity clearance rate was the highest. At a concentration of 40 μg/ml, there was no significant difference in the ability to remove free radicals with 35% elution purity and 50% elution purity, but all were below 75% elution purity, at which point 75% elution purity removes free radicals. The ability is close to Trolox. The above results indicate that the different elution purity of *L. japonica* polyphenols has the ability to scavenge DPPH free radicals and is a good donor of hydrogen protons. However, the ability of different purity of *L. japonica* polyphenols to remove DPPH free radicals is significantly less than that of Trolox.

Past research shows that tyrosinases can catalyze the first two steps of melanin production and are associated with hyperpigmentation disorders such as melasma, solar lentigines, and postinflammatory hyperpigmentation (Fisk, Agbai, Lev‐Tov, & Sivamani, 2014; Xue et al., 2018). Plants that contain natural active ingredients can reduce melanin synthesis through inhibiting tyrosinases and antioxidant effects. Therefore, the application of tyrosinases inhibitors can inhibit pigmentation and achieve whitening effect (Rescigno, Sollai, Pisu, Rinaldi, & Sanjust, 2002). Plant cosmetics have become an increasingly popular alternative to decolorizers because they are safer than standard de‐colorants (Fisk et al., 2014). Tyrosinases cause browning reactions which are unfavorable in fruits and vegetables (Olmedo et al., 2018), but can be slowed by tyrosinases inhibitors (Chang, 2009). In addition, tyrosinase inhibitors can be used as an insect control (Balabanidou et al., 2018; De, Adhikari, Nandy, Saha, & Goswami, 2018; Rezaei, Mohammadi, Mahdavi, Shourian, & Ghafouri, 2018) because the physiological responses of insects, such as the thickening of the stratum corneum or changing the cuticle composition, are related to tyrosinase action. Therefore, the development of safe and effective tyrosinases inhibitors is necessary in medicine, agriculture, cosmetics, and food industry.

It can be seen from the Figure 4 that with the increase in polyphenol concentration, the inhibition rate of tyrosinase of different purity of *L. japonica* polyphenols has a certain upward trend, indicating that the inhibition is continuously enhanced; and at 50–100 μg/ml, the slope of the line is large, indicating that the inhibitory effect on tyrosinase is obvious when the concentration of polyphenol is low, but the inhibition rate is not strong. When the inhibition rate reached 50%, the purity of 30%, 50%, and 75% polyphenols was (355.270 ± 0.84) μg/ml, (282.229) ±1.12) μg/ml, and (205.826 ± 0.23) μg/ml, indicating that the higher the purity of the polyphenol, the better the inhibition effect. The tyrosinase inhibition of *L. japonica* polyphenols may be due to a group of multiple hydroxyl groups on the ring.

In conclusion, shearing time of 3.30 min, ethanol volume fraction of 57%, and a solid–liquid ratio of 1:58 were found to be optimal conditions for the extraction *L. japonica* polyphenols. The polyphenols from *L. japonica* had the effect of scavenging free radicals, antioxidation, antiaging, and whitening. Results from this study validated *L. japonica* as an antioxidant and a tyrosinase inhibitor (Jeong, Jeong, Hwang, & Kim, 2015). The purification effect of macroporous resin was not ideal in this experiment as the monomer substance was unable to be separated and extracted. Subsequent experiments can use HPLC to extract effective monomer substance.

## ETHICAL STATEMENT

This study does not involve any human or animal testing. Written informed consent was obtained from all study participants.

## CONFLICT OF INTEREST

The authors declare that we do not have any conflict of interest.
